# Bis{4-[(*E*)-2-(1*H*-indol-3-yl)ethen­yl]-1-methyl­pyridinium} 4-fluoro­benzene­sulfonate nitrate 0.25-hydrate^1^

**DOI:** 10.1107/S1600536809000026

**Published:** 2009-01-08

**Authors:** Suchada Chantrapromma, Hoong-Kun Fun

**Affiliations:** a Crystal Materials Research Unit, Department of Chemistry, Faculty of Science, Prince of Songkla University, Hat-Yai, Songkhla 90112, Thailand; b X-ray Crystallography Unit, School of Physics, Universiti Sains Malaysia, 11800 USM, Penang, Malaysia

## Abstract

In the title compound, 2C_16_H_15_N_2_^+^·C_6_H_4_FO_3_S^−^·NO_3_^−^·0.25H_2_O, the two cations are nearly planar, with dihedral angles of 1.34 (14) and 4.6 (2)°, respectively, between the pyridinium and indole rings. The cations each adopt *E* configurations with respect to the C=C bonds and are inclined to each other with a dihedral angle of 77.66 (5)°. The ethenyl group of one cation is disordered over two sites with occupancies of 0.685 (12) and 0.315 (12), and the sulfonate group of the 4-fluoro­benzene­sulfonate anion is also disordered with occupancies of 0.535 (10) and 0.465 (10) for the two sets of O atoms. The anion is also inclined to the two cations, with dihedral angles between the mean planes of the benzene ring and the π-conjugated systems of the cations of 24.72 (11) and 79.83 (11)°. In the crystal structure, the cations are stacked in an anti­parallel fashion into columns approximately along the *a* axis and are further linked through the anions into a three-dimensional network *via* N—H⋯O and C—H⋯O inter­actions. The water mol­ecule forms O—H⋯O hydrogen bonds to the nitrate anion and C—H⋯π inter­actions are also observed.

## Related literature

For details of nonlinear optical materials, see, for example: Dittrich *et al.* (2003 ▶); Nogi *et al.* (2000 ▶); Oudar & LePerson (1975 ▶); Sato *et al.* (1999 ▶). For related structures, see, for example: Chantrapromma *et al.* (2006 ▶, 2007 ▶, 2008 ▶). For reference bond-length data, see: Allen *et al.* (1987 ▶).
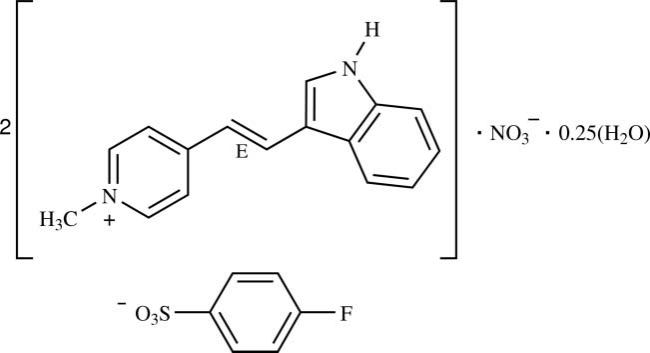


## Experimental

### 

#### Crystal data

2C_16_H_15_N_2_^+^·C_6_H_4_FO_3_S^−^·NO_3_^−^·0.25H_2_O
*M* *_r_* = 712.27
Triclinic, 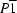

*a* = 8.7750 (6) Å
*b* = 13.6366 (1) Å
*c* = 15.3190 (11) Å
α = 97.520 (1)°
β = 91.236 (1)°
γ = 99.861 (1)°
*V* = 1788.66 (18) Å^3^
*Z* = 2
Mo *K*α radiation
μ = 0.15 mm^−1^
*T* = 297 (2) K
0.57 × 0.39 × 0.12 mm

#### Data collection

Siemens SMART CCD area-detector diffractometer
Absorption correction: empirical (using intensity measurements) (*SADABS*; Sheldrick, 1996 ▶) *T*_min_ = 0.919, *T*_max_ = 0.982
17243 measured reflections
6287 independent reflections
4818 reflections with *I* > 2σ(*I*)
*R*_int_ = 0.018

#### Refinement

*R*[*F* ^2^ > 2σ(*F* ^2^)] = 0.063
*wR*(*F* ^2^) = 0.199
*S* = 1.03
6287 reflections
520 parameters
H-atom parameters constrained
Δρ_max_ = 0.46 e Å^−3^
Δρ_min_ = −0.25 e Å^−3^

### 

Data collection: *SMART* (Siemens, 1996 ▶); cell refinement: *SAINT* (Siemens, 1996 ▶); data reduction: *SAINT*; program(s) used to solve structure: *SHELXTL* (Sheldrick, 2008 ▶); program(s) used to refine structure: *SHELXTL*; molecular graphics: *SHELXTL*; software used to prepare material for publication: *SHELXTL* and *PLATON* (Spek, 2003 ▶).

## Supplementary Material

Crystal structure: contains datablocks global, I. DOI: 10.1107/S1600536809000026/sj2569sup1.cif

Structure factors: contains datablocks I. DOI: 10.1107/S1600536809000026/sj2569Isup2.hkl

Additional supplementary materials:  crystallographic information; 3D view; checkCIF report

## Figures and Tables

**Table 1:** Hydrogen-bond geometry (Å, °) *Cg*1, *Cg*2 and *Cg*3 are the centroids of the rings N4/C30–C32/C37, C32–C37 and C16–C21, respectively.

*D*—H⋯*A*	*D*—H	H⋯*A*	*D*⋯*A*	*D*—H⋯*A*
N2—H1N2⋯O3*A*^i^	0.86	2.09	2.950 (10)	175
N4—H1N4⋯O4^ii^	0.86	2.36	3.106 (4)	145
N4—H1N4⋯O5^ii^	0.86	2.43	3.259 (6)	163
O1*W*—H1*W*1⋯O5^iii^	0.85	2.42	2.711 (14)	101
C6—H6*A*⋯O2*A*	0.93	2.44	2.866 (7)	108
C7—H7*A*⋯O4^iv^	0.93	2.51	3.224 (4)	134
C9—H9*A*⋯O1*A*^v^	0.93	2.47	3.169 (8)	132
C22—H22*A*⋯O1*A*^v^	0.96	2.39	3.256 (8)	149
C22—H22*C*⋯O2*A*^vi^	0.96	2.19	3.147 (8)	175
C23—H23*A*⋯O6^vii^	0.93	2.55	3.456 (5)	164
C15—H15*A*⋯*Cg*1^viii^	0.93	2.99	3.896 (3)	164
C15—H15*A*⋯*Cg*2^viii^	0.93	2.72	3.563 (4)	151
C34—H34*A*⋯*Cg*3^ix^	0.93	2.78	3.595 (4)	147
C38—H38*C*⋯*Cg*2^x^	0.96	2.95	3.695 (4)	135

Symmetry codes: (i) 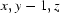; (ii) 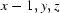; (iii) 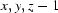; (iv) 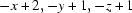; (v) 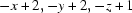; (vi) 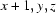; (vii) 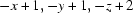; (viii) 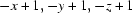; (ix) 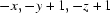; (x) 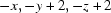.

**Fig. 1.**
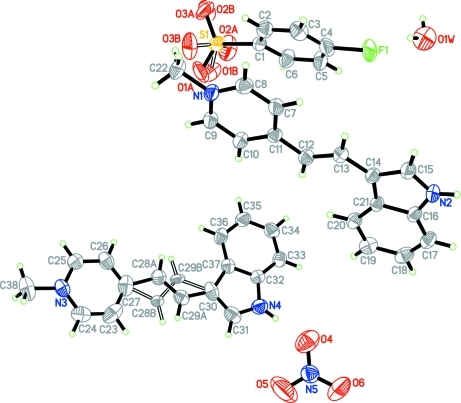
The structure of (I) showing 30% probability displacement ellipsoids and the atom-numbering scheme. Atoms of the major and minor disorder components are joined with solid and open bonds, respectively.

**Fig. 2.**
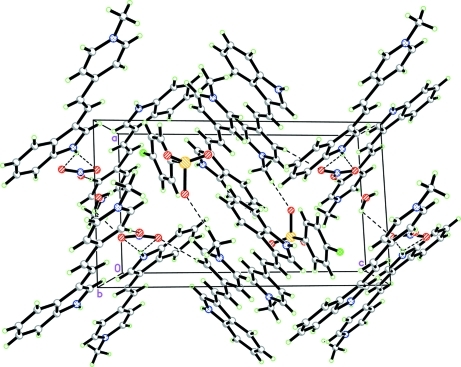
The packing diagram of (I) viewed along the b axis. Hydrogen bonds were drawn as dashed lines and atoms of the minor disorder components are not shown.

## supplementary crystallographic information

### Comment

Much effort has been focused on the development of new materials with nonlinear optical properties. Organic molecules that exhibit second-order nonlinear optical properties usually consist of a framework involving a delocalized π system, end-capped with either a donor or acceptor substituent or both. Several organic compounds such as single crystals of 1-methyl-4-(2-(4-(dimethylamino)phenyl)ethynyl)pyridinium *p*-toluenesulfonate (DAST) and its analogues exhibit second-order nonlinear optical properties (Dittrich *et al.*, 2003; Nogi *et al.*, 2000; Sato *et al.*, 1999). Oudar & LePerson (1975) examined the effect of conjugation length using a stilbene instead of a benzene π-system. In our continuing research on nonlinear optical materials (Chantrapromma *et al.*, 2006, 2007, 2008), the title compound was synthesized as a conjugated π system NLO-chromophore and its crystal structure is reported here.

In the crystal structure of the title compound, the asymmetric unit consists of two C_16_H_15_N_2_^+^ cations, C_6_H_4_FO_3_S^-^ and NO_3_^-^ anions and a solvent water molecule with occupancy approximately 0.25 (Fig. 1). The two cations exist in *E* configurations with respect to the ethenyl unit and have slightly different bond lengths and angles. One cation [C7–C22/N1–N2] is almost planar whereas another cation [C23–C38/N3–N4] is slightly twisted as indicated by the dihedral angles between the pyridinium and indole rings of 1.32 (11)° in the former and 4.72 (16)° in the latter molecule. The ethenyl fragment (C28–C29) of one cation is disordered over two sites with occupancies 0.685 (12) and 0.315 (12) respectively (Fig. 1). The ethenyl unit is nearly planar with respect to the pyridinium and indole rings with the torsion angles C7—C11—C12—C13 = 0.0 (4)°; C12—C13—C14—C15 = -178.6 (3)° [for the C12–C13 ethenyl group] and C26—C27—C28A—C29A = 178.2 (4)°; C28A—C29A—C30—C31 = 176.9 (5)° and C26—C27—C28B—C29B = -3.5 (13)°; C28B—C29B—C30—C31 = -3.8 (13)° [for the C28–C29 ethenyl group]. The π conjugated systems of the two cations are inclined to each other with a dihedral angle of 77.66 (7)°. The sulfonate group of the 4-chlorobenzenesulfonate anion is also disordered with occupancies of 0.535 (10) and 0.465 (10) for the two sets of O atoms (Fig. 1). The anion is inclined to the two cations with dihedral angles between the mean planes of the C1–C6 benzene ring and the π conjugated system of the cations of 24.72 (11)° [for C7–C21/N1–N2] and 79.83 (11)° [for C23–C37/N3–N4]. Bond lengths in the compound are in normal ranges (Allen *et al.*, 1987).

In the crystal structure (Fig. 2), the cations are stacked in an antiparallel fashion into columns approximately along the *a* axis and are further linked by anions into a three-dimensional network *via* N—H···O and C—H···O interactions. Water molecules link to the NO_3_^-^ anions by O—H···O hydrogen bonds (Table 1, Fig. 2). The crystal was stabilized by N—H···O, O—H···O, C—H···O and C—H···π interactions (Table 1); *Cg*1; *Cg*2 and *Cg*3 are the centroids of the N4/C30–C32/C37, C32–C37 and C16–C21 rings, respectively.

### Experimental

4-[(*E*)-2-(1*H*-Indol-3-yl)ethenyl]-1-methylpyridinium iodide (compound A) was synthesized from a mixture (1:1:1 molar ratio) of 1,4-dimethylpyridinium iodide (2.00 g, 8.51 mmol), indole-3-carboxaldehyde (1.24 g, 8.51 mmol) and piperidine (0.84 ml, 8.51 mmol) in methanol (40 ml) under reflux for 2 h under a nitrogen atmosphere. The solid which formed was filtered, washed with ether and recrystallized from methanol to give orange single crystals of compound A after several days. The title compound was synthesized by mixing compound A (0.72 g, 2.0 mmol) in hot methanol (30 ml) and silver(I) 4-fluorobenzenesulfonate (0.57 g, 2.0 mmol) in hot methanol (20 ml). The mixture turned yellow and immediately yielded a gray precipitate of silver iodide. After stirring the mixture for *ca* 30 min, the precipitate of silver iodide was removed and the resulting solution was evaporated to yield an orange solid. Orange block-shaped single crystals of the title compound suitable for X-ray structure determination were recrystallized from methanol by slow evaporation of the solvent at room temperature after several days.

### Refinement

All H atoms were positioned geometrically and allowed to ride on their parent atoms, with O—H = 0.85 Å, N—H = 0.86 Å, C_aryl_—H = 0.93 Å and C_methyl_—H = 0.96 Å. The *U*_iso_ values were constrained to be 1.5*U*_eq_ of the carrier atom for methyl H atoms and 1.2*U*_eq_ for the remaining H atoms. A rotating group model was used for the methyl groups. The highest residual electron density peak is located at 1.02 Å from F1 and the deepest hole is located at 0.95 Å from O4.

The ethenyl group of one cation is disordered over two sites with occupancies 0.685 (12) and 0.315 (12) respectively. A close H···H contact involving a H atom in this disordered group suggests that the disorder should extend to the whole cation molecule. However we were not successful in generating a complete disorder model and could only successfully model the local disorder in the ethenyl fragment of the molecule. The sulfonate group of the 4-fluorobenzenesulfonate anion is also disordered with occupancies of 0.535 (10) and 0.465 (10) for the two sets of O atoms.

### Crystal data

**Table:**

2C_16_H_15_N_2_^+^·C_6_H_4_FO_3_S^−^·NO_3_^−^·0.25H_2_O	*Z* = 2
*M**_r_* = 712.27	*F*(000) = 745
Triclinic, *P*1	*D*_x_ = 1.322 Mg m^−^^3^
Hall symbol: -P 1	Mo *K*α radiation, λ = 0.71073 Å
*a* = 8.7750 (6) Å	Cell parameters from 6287 reflections
*b* = 13.6366 (1) Å	θ = 1.3–25.0°
*c* = 15.3190 (11) Å	µ = 0.15 mm^−^^1^
α = 97.520 (1)°	*T* = 297 K
β = 91.236 (1)°	Plate, orange
γ = 99.861 (1)°	0.57 × 0.39 × 0.12 mm
*V* = 1788.66 (18) Å^3^	

### Data collection

**Table:**

Siemens SMART CCD area-detector diffractometer	6287 independent reflections
Radiation source: fine-focus sealed tube	4818 reflections with *I* > 2σ(*I*)
graphite	*R*_int_ = 0.018
Detector resolution: 8.33 pixels mm^-1^	θ_max_ = 25.0°, θ_min_ = 1.3°
ω scans	*h* = −10→10
Absorption correction: empirical (using intensity measurements) (*SADABS*; Sheldrick, 1996)	*k* = −16→16
*T*_min_ = 0.919, *T*_max_ = 0.982	*l* = −18→18
17243 measured reflections	

### Refinement

**Table:**

Refinement on *F*^2^	Primary atom site location: structure-invariant direct methods
Least-squares matrix: full	Secondary atom site location: difference Fourier map
*R*[*F*^2^ > 2σ(*F*^2^)] = 0.063	Hydrogen site location: inferred from neighbouring sites
*wR*(*F*^2^) = 0.199	H-atom parameters constrained
*S* = 1.03	*w* = 1/[σ^2^(*F*_o_^2^) + (0.1048*P*)^2^ + 0.7458*P*] where *P* = (*F*_o_^2^ + 2*F*_c_^2^)/3
6287 reflections	(Δ/σ)_max_ < 0.001
520 parameters	Δρ_max_ = 0.46 e Å^−^^3^
0 restraints	Δρ_min_ = −0.25 e Å^−^^3^

### Special details

**Table:**

Geometry. All e.s.d.'s (except the e.s.d. in the dihedral angle between two l.s. planes) are estimated using the full covariance matrix. The cell e.s.d.'s are taken into account individually in the estimation of e.s.d.'s in distances, angles and torsion angles; correlations between e.s.d.'s in cell parameters are only used when they are defined by crystal symmetry. An approximate (isotropic) treatment of cell e.s.d.'s is used for estimating e.s.d.'s involving l.s. planes.
Refinement. Refinement of *F*^2^ against ALL reflections. The weighted *R*-factor *wR* and goodness of fit *S* are based on *F*^2^, conventional *R*-factors *R* are based on *F*, with *F* set to zero for negative *F*^2^. The threshold expression of *F*^2^ > σ(*F*^2^) is used only for calculating *R*-factors(gt) *etc*. and is not relevant to the choice of reflections for refinement. *R*-factors based on *F*^2^ are statistically about twice as large as those based on *F*, and *R*- factors based on ALL data will be even larger.

### Fractional atomic coordinates and isotropic or equivalent isotropic displacement parameters (Å^2^)

**Table:**

	*x*	*y*	*z*	*U*_iso_*/*U*_eq_	Occ. (<1)
F1	0.8301 (3)	0.53738 (13)	0.13528 (13)	0.1070 (7)	
S1	0.74535 (14)	0.96215 (6)	0.30690 (6)	0.0943 (4)	
O1A	0.7947 (14)	0.9676 (5)	0.3874 (4)	0.128 (4)	0.535 (10)
O2A	0.5540 (7)	0.9537 (4)	0.3014 (6)	0.122 (3)	0.535 (10)
O3A	0.7858 (15)	1.0287 (7)	0.2507 (7)	0.117 (4)	0.535 (10)
O1B	0.6669 (17)	0.9575 (5)	0.3770 (7)	0.148 (7)	0.465 (10)
O2B	0.7404 (16)	1.0297 (9)	0.2476 (7)	0.106 (4)	0.465 (10)
O3B	0.9324 (9)	1.0071 (5)	0.3454 (6)	0.123 (3)	0.465 (10)
O4	0.7248 (4)	0.3823 (2)	0.8149 (2)	0.1299 (11)	
O5	0.7224 (8)	0.3889 (4)	0.9475 (3)	0.241 (3)	
O6	0.6872 (4)	0.2501 (2)	0.8724 (3)	0.1355 (12)	
N1	1.1622 (3)	0.86382 (16)	0.41062 (15)	0.0630 (6)	
N2	0.7682 (3)	0.22728 (16)	0.34872 (16)	0.0698 (6)	
H1N2	0.7686	0.1677	0.3223	0.084*	
N3	0.4743 (3)	1.1617 (2)	1.09022 (17)	0.0769 (7)	
N4	−0.1419 (3)	0.6053 (2)	0.88513 (19)	0.0872 (8)	
H1N4	−0.1724	0.5431	0.8904	0.105*	
N5	0.7084 (3)	0.3399 (2)	0.8801 (2)	0.0836 (7)	
C1	0.7643 (3)	0.84191 (19)	0.25284 (17)	0.0626 (7)	
C2	0.9019 (4)	0.8268 (2)	0.21795 (19)	0.0722 (8)	
H2A	0.9835	0.8806	0.2195	0.087*	
C3	0.9200 (4)	0.7333 (3)	0.1809 (2)	0.0806 (9)	
H3A	1.0139	0.7239	0.1571	0.097*	
C4	0.8020 (4)	0.6533 (2)	0.1782 (2)	0.0771 (8)	
C5	0.6630 (4)	0.6681 (2)	0.2113 (2)	0.0877 (10)	
H5A	0.5814	0.6142	0.2089	0.105*	
C6	0.6433 (4)	0.7629 (2)	0.2484 (2)	0.0800 (9)	
H6A	0.5483	0.7731	0.2703	0.096*	
C7	1.1090 (3)	0.6898 (2)	0.36194 (18)	0.0651 (7)	
H7A	1.1316	0.6350	0.3248	0.078*	
C8	1.1910 (3)	0.7838 (2)	0.35711 (19)	0.0686 (7)	
H8A	1.2674	0.7922	0.3163	0.082*	
C9	1.0525 (3)	0.8512 (2)	0.46932 (19)	0.0674 (7)	
H9A	1.0334	0.9067	0.5068	0.081*	
C10	0.9685 (3)	0.75962 (19)	0.47560 (19)	0.0646 (7)	
H10A	0.8930	0.7536	0.5172	0.078*	
C11	0.9924 (3)	0.67502 (18)	0.42152 (16)	0.0547 (6)	
C12	0.9001 (3)	0.57771 (19)	0.42901 (17)	0.0595 (6)	
H12A	0.8248	0.5754	0.4708	0.071*	
C13	0.9144 (3)	0.49223 (19)	0.38127 (18)	0.0618 (6)	
H13A	0.9893	0.4968	0.3393	0.074*	
C14	0.8305 (3)	0.39367 (18)	0.38538 (17)	0.0577 (6)	
C15	0.8606 (4)	0.3107 (2)	0.33221 (19)	0.0696 (7)	
H15A	0.9356	0.3124	0.2902	0.084*	
C16	0.6722 (3)	0.25210 (18)	0.41484 (18)	0.0584 (6)	
C17	0.5603 (3)	0.1904 (2)	0.4537 (2)	0.0702 (8)	
H17A	0.5400	0.1213	0.4363	0.084*	
C18	0.4800 (3)	0.2348 (2)	0.5189 (2)	0.0785 (9)	
H18A	0.4033	0.1952	0.5461	0.094*	
C19	0.5115 (3)	0.3387 (2)	0.5452 (2)	0.0762 (8)	
H19A	0.4550	0.3669	0.5894	0.091*	
C20	0.6248 (3)	0.3999 (2)	0.50683 (18)	0.0626 (7)	
H20A	0.6455	0.4688	0.5252	0.075*	
C21	0.7077 (3)	0.35702 (17)	0.44012 (16)	0.0529 (6)	
C22	1.2493 (5)	0.9649 (2)	0.4041 (3)	0.0987 (11)	
H22A	1.2723	1.0015	0.4622	0.148*	
H22B	1.1881	1.0000	0.3705	0.148*	
H22C	1.3441	0.9591	0.3754	0.148*	
C23	0.3467 (6)	0.9948 (3)	1.0839 (4)	0.1117 (15)	
H23A	0.3336	0.9347	1.1075	0.134*	
C24	0.4531 (5)	1.0744 (3)	1.1227 (2)	0.0925 (10)	
H24A	0.5114	1.0677	1.1723	0.111*	
C25	0.3890 (4)	1.1693 (3)	1.0182 (2)	0.0797 (8)	
H25A	0.4030	1.2297	0.9950	0.096*	
C26	0.2841 (4)	1.0915 (3)	0.9792 (2)	0.0863 (10)	
H26A	0.2278	1.0998	0.9293	0.104*	
C27	0.2572 (4)	1.0021 (3)	1.0093 (3)	0.0964 (11)	
C28A	0.1315 (7)	0.9283 (6)	0.9502 (4)	0.077 (2)	0.685 (12)
H28A	0.0848	0.9499	0.9028	0.093*	0.685 (12)
C29A	0.0901 (6)	0.8359 (5)	0.9661 (4)	0.075 (2)	0.685 (12)
H29A	0.1347	0.8176	1.0158	0.090*	0.685 (12)
C28B	0.1802 (10)	0.8954 (7)	1.0036 (6)	0.052 (3)	0.315 (12)
H28B	0.1980	0.8505	1.0419	0.062*	0.315 (12)
C29B	0.0751 (16)	0.8741 (10)	0.9296 (8)	0.057 (3)	0.315 (12)
H29B	0.0611	0.9218	0.8933	0.068*	0.315 (12)
C30	−0.0168 (4)	0.7624 (3)	0.9137 (2)	0.0847 (10)	
C31	−0.0391 (4)	0.6689 (3)	0.9388 (2)	0.0946 (10)	
H31A	0.0111	0.6520	0.9873	0.114*	
C32	−0.1923 (3)	0.6535 (2)	0.81984 (19)	0.0657 (7)	
C33	−0.2938 (3)	0.6159 (2)	0.7491 (2)	0.0756 (8)	
H33A	−0.3421	0.5489	0.7402	0.091*	
C34	−0.3209 (4)	0.6814 (3)	0.6919 (2)	0.0849 (9)	
H34A	−0.3887	0.6584	0.6432	0.102*	
C35	−0.2491 (4)	0.7807 (3)	0.7056 (2)	0.0885 (10)	
H35A	−0.2702	0.8234	0.6660	0.106*	
C36	−0.1480 (4)	0.8182 (2)	0.7758 (2)	0.0803 (9)	
H36A	−0.1004	0.8853	0.7840	0.096*	
C37	−0.1175 (3)	0.7536 (2)	0.83492 (19)	0.0661 (7)	
C38	0.5860 (4)	1.2495 (3)	1.1315 (3)	0.1201 (15)	
H38A	0.6153	1.2939	1.0887	0.180*	
H38B	0.6761	1.2276	1.1532	0.180*	
H38C	0.5393	1.2842	1.1795	0.180*	
O1W	0.4653 (13)	0.4288 (9)	0.0301 (9)	0.128 (4)	0.25
H1W1	0.5529	0.4619	0.0500	0.192*	0.25
H2W1	0.4351	0.4572	−0.0116	0.192*	0.25

### Atomic displacement parameters (Å^2^)

**Table:**

	*U*^11^	*U*^22^	*U*^33^	*U*^12^	*U*^13^	*U*^23^
F1	0.168 (2)	0.0544 (10)	0.1004 (14)	0.0473 (11)	0.0094 (13)	−0.0183 (9)
S1	0.1704 (10)	0.0472 (4)	0.0706 (5)	0.0300 (5)	0.0304 (6)	0.0093 (3)
O1A	0.230 (11)	0.084 (4)	0.065 (4)	0.053 (7)	−0.043 (6)	−0.029 (3)
O2A	0.100 (4)	0.093 (4)	0.177 (7)	0.043 (3)	0.034 (4)	−0.013 (4)
O3A	0.190 (9)	0.040 (3)	0.126 (7)	0.019 (4)	0.081 (6)	0.027 (4)
O1B	0.252 (14)	0.067 (4)	0.139 (10)	0.049 (7)	0.138 (11)	0.020 (5)
O2B	0.168 (8)	0.079 (5)	0.078 (5)	0.062 (5)	−0.045 (6)	−0.007 (4)
O3B	0.114 (5)	0.083 (4)	0.153 (6)	0.002 (4)	−0.037 (5)	−0.029 (4)
O4	0.135 (2)	0.110 (2)	0.156 (3)	0.0253 (18)	0.055 (2)	0.045 (2)
O5	0.396 (8)	0.236 (5)	0.098 (3)	0.138 (5)	−0.060 (4)	−0.045 (3)
O6	0.124 (2)	0.086 (2)	0.203 (3)	0.0166 (17)	−0.014 (2)	0.050 (2)
N1	0.0650 (13)	0.0505 (12)	0.0690 (14)	−0.0022 (10)	−0.0069 (11)	0.0094 (10)
N2	0.0892 (16)	0.0444 (12)	0.0731 (15)	0.0114 (11)	0.0006 (13)	−0.0007 (10)
N3	0.0627 (14)	0.0909 (19)	0.0725 (15)	0.0172 (13)	0.0045 (12)	−0.0102 (14)
N4	0.0919 (19)	0.0847 (18)	0.0885 (18)	0.0187 (15)	−0.0002 (15)	0.0213 (15)
N5	0.0857 (18)	0.087 (2)	0.0799 (19)	0.0249 (15)	−0.0081 (14)	0.0076 (16)
C1	0.0860 (19)	0.0502 (14)	0.0534 (14)	0.0155 (13)	0.0096 (13)	0.0077 (11)
C2	0.0700 (18)	0.0675 (18)	0.0755 (18)	0.0049 (14)	0.0007 (14)	0.0067 (14)
C3	0.0766 (19)	0.088 (2)	0.081 (2)	0.0330 (18)	0.0066 (16)	0.0031 (17)
C4	0.103 (2)	0.0663 (18)	0.0633 (17)	0.0322 (17)	−0.0080 (16)	−0.0074 (14)
C5	0.090 (2)	0.0594 (18)	0.103 (2)	−0.0051 (16)	0.0003 (19)	−0.0035 (16)
C6	0.0766 (19)	0.0626 (18)	0.100 (2)	0.0125 (15)	0.0224 (17)	0.0054 (16)
C7	0.0765 (17)	0.0536 (15)	0.0646 (16)	0.0145 (13)	−0.0014 (13)	0.0022 (12)
C8	0.0648 (16)	0.0740 (18)	0.0664 (17)	0.0049 (14)	0.0057 (13)	0.0167 (14)
C9	0.0754 (18)	0.0527 (15)	0.0713 (17)	0.0084 (13)	0.0003 (14)	0.0022 (13)
C10	0.0665 (16)	0.0540 (15)	0.0731 (17)	0.0088 (12)	0.0069 (13)	0.0093 (13)
C11	0.0557 (14)	0.0518 (13)	0.0574 (14)	0.0090 (11)	−0.0035 (11)	0.0118 (11)
C12	0.0600 (14)	0.0564 (15)	0.0632 (15)	0.0117 (12)	0.0060 (12)	0.0092 (12)
C13	0.0671 (16)	0.0531 (14)	0.0650 (16)	0.0083 (12)	0.0042 (12)	0.0101 (12)
C14	0.0625 (15)	0.0491 (13)	0.0623 (15)	0.0114 (11)	0.0002 (12)	0.0088 (11)
C15	0.0844 (19)	0.0546 (15)	0.0704 (17)	0.0140 (14)	0.0131 (14)	0.0063 (13)
C16	0.0588 (14)	0.0470 (13)	0.0687 (16)	0.0062 (11)	−0.0120 (12)	0.0118 (11)
C17	0.0646 (16)	0.0535 (15)	0.090 (2)	0.0000 (13)	−0.0104 (15)	0.0178 (14)
C18	0.0594 (16)	0.078 (2)	0.099 (2)	−0.0031 (14)	0.0003 (16)	0.0362 (18)
C19	0.0650 (17)	0.083 (2)	0.085 (2)	0.0186 (15)	0.0117 (15)	0.0205 (16)
C20	0.0607 (15)	0.0543 (14)	0.0749 (17)	0.0139 (12)	0.0018 (13)	0.0114 (13)
C21	0.0534 (13)	0.0437 (12)	0.0616 (14)	0.0092 (10)	−0.0073 (11)	0.0080 (10)
C22	0.111 (3)	0.0634 (19)	0.108 (3)	−0.0272 (18)	−0.002 (2)	0.0177 (18)
C23	0.123 (3)	0.077 (2)	0.151 (4)	0.038 (2)	0.073 (3)	0.034 (3)
C24	0.100 (3)	0.112 (3)	0.081 (2)	0.050 (2)	0.0186 (19)	0.025 (2)
C25	0.081 (2)	0.080 (2)	0.081 (2)	0.0189 (16)	0.0132 (17)	0.0112 (16)
C26	0.0677 (19)	0.104 (3)	0.080 (2)	0.0116 (18)	−0.0010 (16)	−0.0069 (19)
C27	0.076 (2)	0.088 (3)	0.118 (3)	0.0099 (19)	0.033 (2)	−0.014 (2)
C28A	0.085 (4)	0.074 (4)	0.076 (4)	0.023 (3)	0.010 (3)	0.006 (3)
C29A	0.075 (4)	0.086 (4)	0.066 (3)	0.027 (3)	0.007 (3)	0.003 (3)
C28B	0.056 (6)	0.052 (5)	0.050 (5)	0.011 (4)	−0.001 (4)	0.014 (4)
C29B	0.084 (8)	0.055 (7)	0.045 (6)	0.033 (6)	0.016 (5)	0.025 (5)
C30	0.0618 (18)	0.099 (3)	0.084 (2)	0.0144 (17)	0.0064 (16)	−0.0212 (19)
C31	0.094 (2)	0.110 (3)	0.081 (2)	0.032 (2)	−0.0093 (19)	0.002 (2)
C32	0.0613 (15)	0.0689 (17)	0.0699 (17)	0.0166 (13)	0.0105 (13)	0.0125 (14)
C33	0.0676 (17)	0.0706 (18)	0.086 (2)	0.0096 (14)	0.0013 (15)	0.0058 (16)
C34	0.082 (2)	0.096 (2)	0.078 (2)	0.0226 (18)	−0.0103 (16)	0.0067 (18)
C35	0.106 (3)	0.076 (2)	0.091 (2)	0.0263 (19)	0.007 (2)	0.0215 (18)
C36	0.081 (2)	0.0619 (17)	0.097 (2)	0.0138 (15)	0.0205 (18)	0.0027 (16)
C37	0.0591 (15)	0.0723 (18)	0.0669 (16)	0.0160 (13)	0.0166 (13)	0.0017 (14)
C38	0.083 (2)	0.132 (3)	0.124 (3)	0.006 (2)	0.000 (2)	−0.044 (3)
O1W	0.091 (7)	0.134 (10)	0.159 (11)	0.018 (7)	0.018 (7)	0.022 (8)

### Geometric parameters (Å, °)

**Table:**

F1—C4	1.692 (3)	C16—C17	1.378 (4)
S1—O1A	1.287 (6)	C16—C21	1.411 (3)
S1—O1B	1.291 (6)	C17—C18	1.371 (4)
S1—O3A	1.340 (8)	C17—H17A	0.9300
S1—O2B	1.381 (11)	C18—C19	1.398 (4)
S1—O2A	1.662 (6)	C18—H18A	0.9300
S1—O3B	1.714 (7)	C19—C20	1.378 (4)
S1—C1	1.774 (3)	C19—H19A	0.9300
O4—N5	1.217 (4)	C20—C21	1.392 (4)
O5—N5	1.145 (4)	C20—H20A	0.9300
O6—N5	1.197 (4)	C22—H22A	0.9600
N1—C9	1.336 (4)	C22—H22B	0.9600
N1—C8	1.340 (4)	C22—H22C	0.9600
N1—C22	1.474 (3)	C23—C24	1.365 (6)
N2—C15	1.336 (4)	C23—C27	1.396 (6)
N2—C16	1.376 (4)	C23—H23A	0.9300
N2—H1N2	0.8600	C24—H24A	0.9300
N3—C24	1.336 (5)	C25—C26	1.345 (5)
N3—C25	1.344 (4)	C25—H25A	0.9300
N3—C38	1.471 (4)	C26—C27	1.345 (5)
N4—C31	1.324 (4)	C26—H26A	0.9300
N4—C32	1.371 (4)	C27—C28B	1.487 (10)
N4—H1N4	0.8600	C27—C28A	1.546 (8)
C1—C2	1.368 (4)	C28A—C29A	1.306 (12)
C1—C6	1.370 (4)	C28A—H28A	0.9300
C2—C3	1.363 (4)	C29A—C30	1.402 (7)
C2—H2A	0.9300	C29A—H29A	0.9300
C3—C4	1.365 (5)	C28B—C29B	1.41 (2)
C3—H3A	0.9300	C28B—H28B	0.9300
C4—C5	1.367 (5)	C29B—C30	1.583 (15)
C5—C6	1.383 (4)	C29B—H29B	0.9300
C5—H5A	0.9300	C30—C31	1.364 (5)
C6—H6A	0.9300	C30—C37	1.459 (4)
C7—C8	1.370 (4)	C31—H31A	0.9300
C7—C11	1.392 (4)	C32—C33	1.374 (4)
C7—H7A	0.9300	C32—C37	1.397 (4)
C8—H8A	0.9300	C33—C34	1.376 (4)
C9—C10	1.353 (4)	C33—H33A	0.9300
C9—H9A	0.9300	C34—C35	1.379 (5)
C10—C11	1.379 (4)	C34—H34A	0.9300
C10—H10A	0.9300	C35—C36	1.364 (5)
C11—C12	1.451 (3)	C35—H35A	0.9300
C12—C13	1.319 (4)	C36—C37	1.396 (4)
C12—H12A	0.9300	C36—H36A	0.9300
C13—C14	1.428 (4)	C38—H38A	0.9600
C13—H13A	0.9300	C38—H38B	0.9600
C14—C15	1.373 (4)	C38—H38C	0.9600
C14—C21	1.441 (4)	O1W—H1W1	0.8500
C15—H15A	0.9300	O1W—H2W1	0.8500
			
O1A—S1—O1B	51.1 (5)	C18—C17—C16	117.4 (3)
O1A—S1—O3A	127.5 (7)	C18—C17—H17A	121.3
O1B—S1—O3A	137.3 (6)	C16—C17—H17A	121.3
O1A—S1—O2B	135.5 (6)	C17—C18—C19	121.1 (3)
O1B—S1—O2B	125.9 (7)	C17—C18—H18A	119.4
O1A—S1—O2A	110.4 (6)	C19—C18—H18A	119.4
O1B—S1—O2A	59.4 (6)	C20—C19—C18	121.2 (3)
O3A—S1—O2A	100.2 (6)	C20—C19—H19A	119.4
O2B—S1—O2A	83.2 (6)	C18—C19—H19A	119.4
O1A—S1—O3B	55.1 (5)	C19—C20—C21	119.0 (3)
O1B—S1—O3B	104.6 (7)	C19—C20—H20A	120.5
O3A—S1—O3B	80.9 (6)	C21—C20—H20A	120.5
O2B—S1—O3B	96.4 (6)	C20—C21—C16	118.3 (2)
O2A—S1—O3B	157.3 (3)	C20—C21—C14	135.6 (2)
O1A—S1—C1	107.2 (3)	C16—C21—C14	106.1 (2)
O1B—S1—C1	112.7 (3)	N1—C22—H22A	109.5
O3A—S1—C1	107.6 (5)	N1—C22—H22B	109.5
O2B—S1—C1	111.5 (5)	H22A—C22—H22B	109.5
O2A—S1—C1	100.7 (2)	N1—C22—H22C	109.5
O3B—S1—C1	100.5 (2)	H22A—C22—H22C	109.5
C9—N1—C8	119.3 (2)	H22B—C22—H22C	109.5
C9—N1—C22	120.3 (3)	C24—C23—C27	121.2 (4)
C8—N1—C22	120.4 (3)	C24—C23—H23A	119.4
C15—N2—C16	109.0 (2)	C27—C23—H23A	119.4
C15—N2—H1N2	125.5	N3—C24—C23	120.7 (4)
C16—N2—H1N2	125.5	N3—C24—H24A	119.6
C24—N3—C25	118.5 (3)	C23—C24—H24A	119.6
C24—N3—C38	122.0 (4)	N3—C25—C26	121.5 (3)
C25—N3—C38	119.5 (3)	N3—C25—H25A	119.3
C31—N4—C32	109.9 (3)	C26—C25—H25A	119.3
C31—N4—H1N4	125.0	C27—C26—C25	122.6 (4)
C32—N4—H1N4	125.0	C27—C26—H26A	118.7
O5—N5—O6	122.5 (5)	C25—C26—H26A	118.7
O5—N5—O4	117.6 (5)	C26—C27—C23	115.6 (4)
O6—N5—O4	119.8 (4)	C26—C27—C28B	152.5 (6)
C2—C1—C6	119.6 (3)	C23—C27—C28B	91.9 (5)
C2—C1—S1	120.0 (2)	C26—C27—C28A	110.3 (5)
C6—C1—S1	120.3 (2)	C23—C27—C28A	134.2 (5)
C3—C2—C1	120.3 (3)	C29A—C28A—C27	120.6 (6)
C3—C2—H2A	119.9	C29A—C28A—H28A	119.7
C1—C2—H2A	119.9	C27—C28A—H28A	119.7
C2—C3—C4	120.8 (3)	C28A—C29A—C30	124.6 (7)
C2—C3—H3A	119.6	C28A—C29A—H29A	117.7
C4—C3—H3A	119.6	C30—C29A—H29A	117.7
C3—C4—C5	119.3 (3)	C29B—C28B—C27	107.8 (9)
C3—C4—F1	119.7 (3)	C29B—C28B—H28B	126.1
C5—C4—F1	120.9 (3)	C27—C28B—H28B	126.1
C4—C5—C6	120.2 (3)	C28B—C29B—C30	114.3 (10)
C4—C5—H5A	119.9	C28B—C29B—H29B	122.8
C6—C5—H5A	119.9	C30—C29B—H29B	122.8
C1—C6—C5	119.7 (3)	C31—C30—C29A	115.9 (5)
C1—C6—H6A	120.1	C31—C30—C37	105.4 (3)
C5—C6—H6A	120.1	C29A—C30—C37	138.7 (5)
C8—C7—C11	121.0 (3)	C31—C30—C29B	147.6 (6)
C8—C7—H7A	119.5	C37—C30—C29B	106.9 (6)
C11—C7—H7A	119.5	N4—C31—C30	111.1 (3)
N1—C8—C7	120.7 (3)	N4—C31—H31A	124.5
N1—C8—H8A	119.7	C30—C31—H31A	124.5
C7—C8—H8A	119.7	N4—C32—C33	129.5 (3)
N1—C9—C10	121.7 (3)	N4—C32—C37	107.6 (3)
N1—C9—H9A	119.2	C33—C32—C37	122.8 (3)
C10—C9—H9A	119.2	C32—C33—C34	117.2 (3)
C9—C10—C11	121.3 (3)	C32—C33—H33A	121.4
C9—C10—H10A	119.3	C34—C33—H33A	121.4
C11—C10—H10A	119.3	C33—C34—C35	121.0 (3)
C10—C11—C7	115.9 (2)	C33—C34—H34A	119.5
C10—C11—C12	120.4 (2)	C35—C34—H34A	119.5
C7—C11—C12	123.7 (2)	C36—C35—C34	121.9 (3)
C13—C12—C11	125.3 (3)	C36—C35—H35A	119.1
C13—C12—H12A	117.4	C34—C35—H35A	119.1
C11—C12—H12A	117.4	C35—C36—C37	118.6 (3)
C12—C13—C14	128.8 (3)	C35—C36—H36A	120.7
C12—C13—H13A	115.6	C37—C36—H36A	120.7
C14—C13—H13A	115.6	C36—C37—C32	118.5 (3)
C15—C14—C13	122.2 (3)	C36—C37—C30	135.4 (3)
C15—C14—C21	105.9 (2)	C32—C37—C30	106.0 (3)
C13—C14—C21	131.9 (2)	N3—C38—H38A	109.5
N2—C15—C14	111.1 (3)	N3—C38—H38B	109.5
N2—C15—H15A	124.4	H38A—C38—H38B	109.5
C14—C15—H15A	124.4	N3—C38—H38C	109.5
N2—C16—C17	129.2 (3)	H38A—C38—H38C	109.5
N2—C16—C21	107.9 (2)	H38B—C38—H38C	109.5
C17—C16—C21	123.0 (3)	H1W1—O1W—H2W1	107.7
			
O1A—S1—C1—C2	−85.3 (6)	C15—C14—C21—C20	179.7 (3)
O1B—S1—C1—C2	−139.6 (8)	C13—C14—C21—C20	0.6 (5)
O3A—S1—C1—C2	54.9 (6)	C15—C14—C21—C16	−0.2 (3)
O2B—S1—C1—C2	72.4 (7)	C13—C14—C21—C16	−179.2 (3)
O2A—S1—C1—C2	159.3 (4)	C25—N3—C24—C23	−0.3 (5)
O3B—S1—C1—C2	−28.8 (4)	C38—N3—C24—C23	178.8 (3)
O1A—S1—C1—C6	92.5 (6)	C27—C23—C24—N3	0.0 (5)
O1B—S1—C1—C6	38.2 (8)	C24—N3—C25—C26	0.1 (4)
O3A—S1—C1—C6	−127.4 (7)	C38—N3—C25—C26	−179.0 (3)
O2B—S1—C1—C6	−109.8 (7)	N3—C25—C26—C27	0.3 (5)
O2A—S1—C1—C6	−23.0 (4)	C25—C26—C27—C23	−0.5 (5)
O3B—S1—C1—C6	149.0 (4)	C25—C26—C27—C28B	−175.9 (8)
C6—C1—C2—C3	−1.6 (5)	C25—C26—C27—C28A	−179.6 (3)
S1—C1—C2—C3	176.2 (2)	C24—C23—C27—C26	0.4 (5)
C1—C2—C3—C4	−0.3 (5)	C24—C23—C27—C28B	178.2 (4)
C2—C3—C4—C5	1.6 (5)	C24—C23—C27—C28A	179.2 (4)
C2—C3—C4—F1	−177.7 (2)	C26—C27—C28A—C29A	178.2 (4)
C3—C4—C5—C6	−1.1 (5)	C23—C27—C28A—C29A	−0.7 (8)
F1—C4—C5—C6	178.2 (3)	C28B—C27—C28A—C29A	0.7 (5)
C2—C1—C6—C5	2.1 (5)	C27—C28A—C29A—C30	−176.6 (4)
S1—C1—C6—C5	−175.7 (3)	C26—C27—C28B—C29B	−3.5 (13)
C4—C5—C6—C1	−0.8 (5)	C23—C27—C28B—C29B	−179.3 (6)
C9—N1—C8—C7	0.2 (4)	C28A—C27—C28B—C29B	1.7 (6)
C22—N1—C8—C7	−179.1 (3)	C27—C28B—C29B—C30	179.7 (6)
C11—C7—C8—N1	0.9 (4)	C28A—C29A—C30—C31	176.9 (5)
C8—N1—C9—C10	−0.7 (4)	C28A—C29A—C30—C37	−2.4 (8)
C22—N1—C9—C10	178.6 (3)	C28A—C29A—C30—C29B	0.9 (8)
N1—C9—C10—C11	0.0 (4)	C28B—C29B—C30—C31	−3.8 (13)
C9—C10—C11—C7	1.0 (4)	C28B—C29B—C30—C29A	2.9 (5)
C9—C10—C11—C12	−179.6 (2)	C28B—C29B—C30—C37	−179.3 (6)
C8—C7—C11—C10	−1.4 (4)	C32—N4—C31—C30	0.9 (4)
C8—C7—C11—C12	179.1 (2)	C29A—C30—C31—N4	−180.0 (3)
C10—C11—C12—C13	−179.4 (3)	C37—C30—C31—N4	−0.5 (4)
C7—C11—C12—C13	0.0 (4)	C29B—C30—C31—N4	−176.0 (7)
C11—C12—C13—C14	178.9 (2)	C31—N4—C32—C33	177.5 (3)
C12—C13—C14—C15	−178.6 (3)	C31—N4—C32—C37	−0.8 (4)
C12—C13—C14—C21	0.4 (5)	N4—C32—C33—C34	−178.3 (3)
C16—N2—C15—C14	−0.3 (3)	C37—C32—C33—C34	−0.2 (4)
C13—C14—C15—N2	179.5 (2)	C32—C33—C34—C35	−0.2 (5)
C21—C14—C15—N2	0.3 (3)	C33—C34—C35—C36	0.4 (5)
C15—N2—C16—C17	−179.1 (3)	C34—C35—C36—C37	−0.1 (5)
C15—N2—C16—C21	0.2 (3)	C35—C36—C37—C32	−0.2 (4)
N2—C16—C17—C18	−180.0 (3)	C35—C36—C37—C30	177.6 (3)
C21—C16—C17—C18	0.8 (4)	N4—C32—C37—C36	178.9 (3)
C16—C17—C18—C19	−0.4 (4)	C33—C32—C37—C36	0.4 (4)
C17—C18—C19—C20	−0.3 (5)	N4—C32—C37—C30	0.5 (3)
C18—C19—C20—C21	0.6 (4)	C33—C32—C37—C30	−178.0 (3)
C19—C20—C21—C16	−0.2 (4)	C31—C30—C37—C36	−178.0 (3)
C19—C20—C21—C14	180.0 (3)	C29A—C30—C37—C36	1.3 (7)
N2—C16—C21—C20	−179.9 (2)	C29B—C30—C37—C36	−0.5 (6)
C17—C16—C21—C20	−0.5 (4)	C31—C30—C37—C32	0.0 (3)
N2—C16—C21—C14	0.0 (3)	C29A—C30—C37—C32	179.3 (4)
C17—C16—C21—C14	179.4 (2)	C29B—C30—C37—C32	177.5 (4)

### Hydrogen-bond geometry (Å, °)

**Table:**

*D*—H···*A*	*D*—H	H···*A*	*D*···*A*	*D*—H···*A*
N2—H1N2···O3A^i^	0.86	2.09	2.950 (10)	175
N4—H1N4···O4^ii^	0.86	2.36	3.106 (4)	145
N4—H1N4···O5^ii^	0.86	2.43	3.259 (6)	163
O1W—H1W1···O5^iii^	0.85	2.42	2.711 (14)	101
C6—H6A···O2A	0.93	2.44	2.866 (7)	108
C7—H7A···O4^iv^	0.93	2.51	3.224 (4)	134
C9—H9A···O1A^v^	0.93	2.47	3.169 (8)	132
C22—H22A···O1A^v^	0.96	2.39	3.256 (8)	149
C22—H22C···O2A^vi^	0.96	2.19	3.147 (8)	175
C23—H23A···O6^vii^	0.93	2.55	3.456 (5)	164
C15—H15A···Cg1^viii^	0.93	2.99	3.896 (3)	164
C15—H15A···Cg2^viii^	0.93	2.72	3.563 (4)	151
C34—H34A···Cg3^ix^	0.93	2.78	3.595 (4)	147
C38—H38C···Cg2^x^	0.96	2.95	3.695 (4)	135

Symmetry codes: (i) *x*, *y*−1, *z*; (ii) *x*−1, *y*, *z*; (iii) *x*, *y*, *z*−1;  (iv) −*x*+2, −*y*+1, −*z*+1; (v) −*x*+2, −*y*+2, −*z*+1; (vi) *x*+1, *y*, *z*; (vii) −*x*+1, −*y*+1, −*z*+2; (viii) −*x*+1, −*y*+1, −*z*+1; (ix) −*x*, −*y*+1, −*z*+1; (x) −*x*, −*y*+2, −*z*+2.
